# Retrospective Evaluation of New Chinese Diagnostic Scoring System for Disseminated Intravascular Coagulation

**DOI:** 10.1371/journal.pone.0129170

**Published:** 2015-06-15

**Authors:** Manzhi Wang, Haiming Kou, Jun Deng, Huafang Wang, Tao Guo, Heng Mei, Yu Hu

**Affiliations:** Institute of Hematology, Union Hospital, Huazhong University of Science and Technology, Wuhan, China; Northwestern University, UNITED STATES

## Abstract

**Objectives:**

To retrospectively validate the new Chinese DIC scoring system (CDSS).

**Methods:**

This study retrospectively collected the information of 619 patients (371 cases with non-hematologic malignancies, 248 cases with hematologic malignancies) who suspected of DIC in Wuhan Union Hospital during 2013-4 to 2014-6. We validated CDSS by comparing it with three leading scoring systems, from International Society on Thrombosis and Haemostasis (ISTH), Japanese Association for Acute Medicine (JAAM) and Japanese Ministry of Health and Welfare (JMHW), and evaluated its prognostic value by 28 days mortality, APACHE II and SOFA score.

**Results:**

In non-hematologic malignancies, CDSS was more specific than JAAM (72.55% vs. 50.49%, p<0.05) and more sensitive than ISTH (77.07% vs. 62.03%, p<0.05). In hematologic malignancies, the area under the ROC curve of CDSS was larger than ISTH and JMHW (0.933 vs. 0.889, p<0.01 with ISTH, 0.944 vs. 0.845, p<0.01 with JMHW). In addition, the 28-day mortality rate, SOFA scores, APACHE II scores of DIC patients diagnosed by CDSS were significantly greater than non-DIC (P <0.05).

**Conclusions:**

We are the first group to propose CDSS. It emphasized the values of the clinical manifestations, the rapidly declining platelet count, APTT in the diagnosis of DIC and used D-dimer as the fibrin-related maker. DIC with hematological malignancies was treated as a special part. In this study we can see that CDSS displayed an acceptable property for the diagnosis of DIC with appropriate sensitivity and specificity, and also had a good prognostic value for DIC patients.

## Introduction

Disseminated intravascular coagulation (DIC) is an acquired syndrome rather than a disease in itself. It is always secondary to an underlying disorder which causes the activation of coagulation. The development of DIC often involves multiple systems such as coagulation system, anticoagulation system and fibrinolytic system [1]. The spectrum of clinical manifestations of DIC includes bleeding, microcirculatory disorder, micro vascular thrombosis and microangiopathic hemolysis[2]. Therefore, DIC is complicated and no single clinical or laboratory test has an adequate sensitivity and specificity to confirm or reject a diagnosis of DIC [3]. It is still a challenging work needing abundant professional experience to diagnose DIC accurately.

In the diagnosis of DIC, underlying disorders and clinical manifestations are two very important parts and laboratory tests are also combined in a scoring algorithm. Molecular markers that are more sensitive for coagulation activation are usually insufficiently specific and are often not available in most settings for daily clinical care [4]. Combinations of routinely available coagulation tests, like platelet count, prothrombin time (PT), a fibrin-related marker and fibrinogen remain the cornerstone of diagnosis [3]. In addition, DIC is a dynamic pathological process while laboratory tests only reflect one moment in the process, so "dynamic monitoring" is more helpful for DIC diagnosis [2].

In China, as early as 1986, the first DIC diagnostic criteria had been proposed [5]. The revised one proposed in the Eighth National Conference of Thrombosis and Haemostasis (2001, Wuhan) [6] had been generally accepted and applied in clinical applications. In 2012, Chinese experts conducted a revise and the latest 2012 Chinese experts’ consensus on the diagnosis of DIC [7] appeared, but there still existed the defect of precise quantification [2]. In recent years, the West and Japanese experts have put forward guidelines or consensus for the diagnosis and treatment of DIC. Currently there are three leading DIC diagnostic criteria used internationally: ISTH (International Society on Thrombosis and Haemostasis) [8], JMHW (Japanese Ministry of Health and Welfare) [9], JAAM (Japanese Association of Acute Medicine) [10]. Many prospective studies had been carried out to compare their diagnostic efficacy, but the consequences remained controversial [3, 10–15].

In summary, on the basis of the analysis of retrospective data and international leading scoring systems, we quantified the Chinese experts’ consensus (2012), and proposed Chinese DIC scoring system (CDSS) (Table 1). This study would retrospectively validate CDSS in patients suspected of DIC.

**Table 1:** Chinese DIC Scoring System (CDSS).

Items	Score
1.Uderlying disease	2
2.Clinical manifestations	
(1)abnormal bleeding independent of original disease*	meet anyone
(2)shock or microcirculatory disorder independent of original disease	1
(3)unexplained organ failure	
3.Laboratory examinations	
(1)Platelet count*(10^9^/L)	
≥100	0
80–100	1
<80	2
≥50% decrease within 24 hrs	1
(2)D-Dimer(mg/L)	
<5	0
5–9	2
≥9	3
(3)Prolongation of PT and APTT	
Prolongation of PT<3s and prolongation of APTT<10s	0
Prolongation of PT≥3s or prolongation of APTT≥10s	1
Prolongation of PT≥6s	2
(4)Fibrinogen(g/L)	
≥1.0	0
<1.0	1

Diagnosis: Seven points or more.

*If underlying disease is hematological malignancy:1.ignore item(1) from Clinical manifestations; 2.Platelet count: PLT<50×10^9^/l:1point,≥50% decrease within 24 hrs:1point; 3.Diagnosis: Six points or more. PT, prothrombin time. APTT, activated partial thromboplastin time.

## Methods and Analysis

### Patients

We retrospectively collected the information of 619 patients who were inpatients suspected of DIC in Wuhan union hospital during 2013–4 to 2014-6.The male-to-female ratio was 342:277, and 248 patients were hematologic malignancies, while 371 non-hematologic malignancies.

The inclusion criteria [11, 15] were the observation of more than one abnormal finding according to the laboratory tests: platelet count< 120×10^9^/l, fibrin/fibrinogen degradation products (FDP) ≥10 mg/l, fibrinogen < 1 g/l, prolongation of prothrombin time (PT) ≥3s. Patients with hematologic malignancies needed to meet at least one of the last three.

Patients who met the following criteria were excluded [3, 10, 11, 13–15]:<15 yrs of age, liver cirrhosis classified as Child-Pugh grade C, known clotting disorders or receipt of anticoagulant therapy, thrombotic thrombocytopenic purpura (TTP), antiphospholipid syndrome (APS, diagnosed according to the Sapporo criteria[16]) et al.

### Ethics statement

In this retrospective cross-sectional study, patients were treated with standard treatment protocol and before starting transfusion of blood products, written informed consent forms were signed by patients. This study was approved by ethics committee of Tongji Medical College, Huazhong University of Science and Technology. The ethics committee waived written informed consent of patients included in the study because this retrospective study just collected the clinical and laboratory data of patients anonymously.

### Method

Due to the specificity of DIC with hematologic malignancies, we analyzed its data statistically by itself. DIC was diagnosed by ISTH, JMHW, JAAM and CDSS. Bleeding symptoms and organ dysfunction in the JMHW criteria were defined as abnormal bleeding independent of original diseases and SOFA score [17] ≥ 2 respectively [10]. FDP measurement was used for the fibrin-related marker in the ISTH criteria, No increase, moderate increase, and strong increase were defined as FDP values of <10, 10≤FDP<25, and ≥25 mg/l [10, 14]. The rapidly declining platelet count in CDSS was defined as a decrease in platelet count of ≥50% per 24 hrs. We used Systemic Inflammation Response Syndrome (SIRS) score [18] to assess systemic inflammatory state, APACHE II score [19] to assess the severity of disease, SOFA score [17] to assess organ dysfunction (The coagulation score was not included in assessment of the SOFA score).

**Fig 1:**
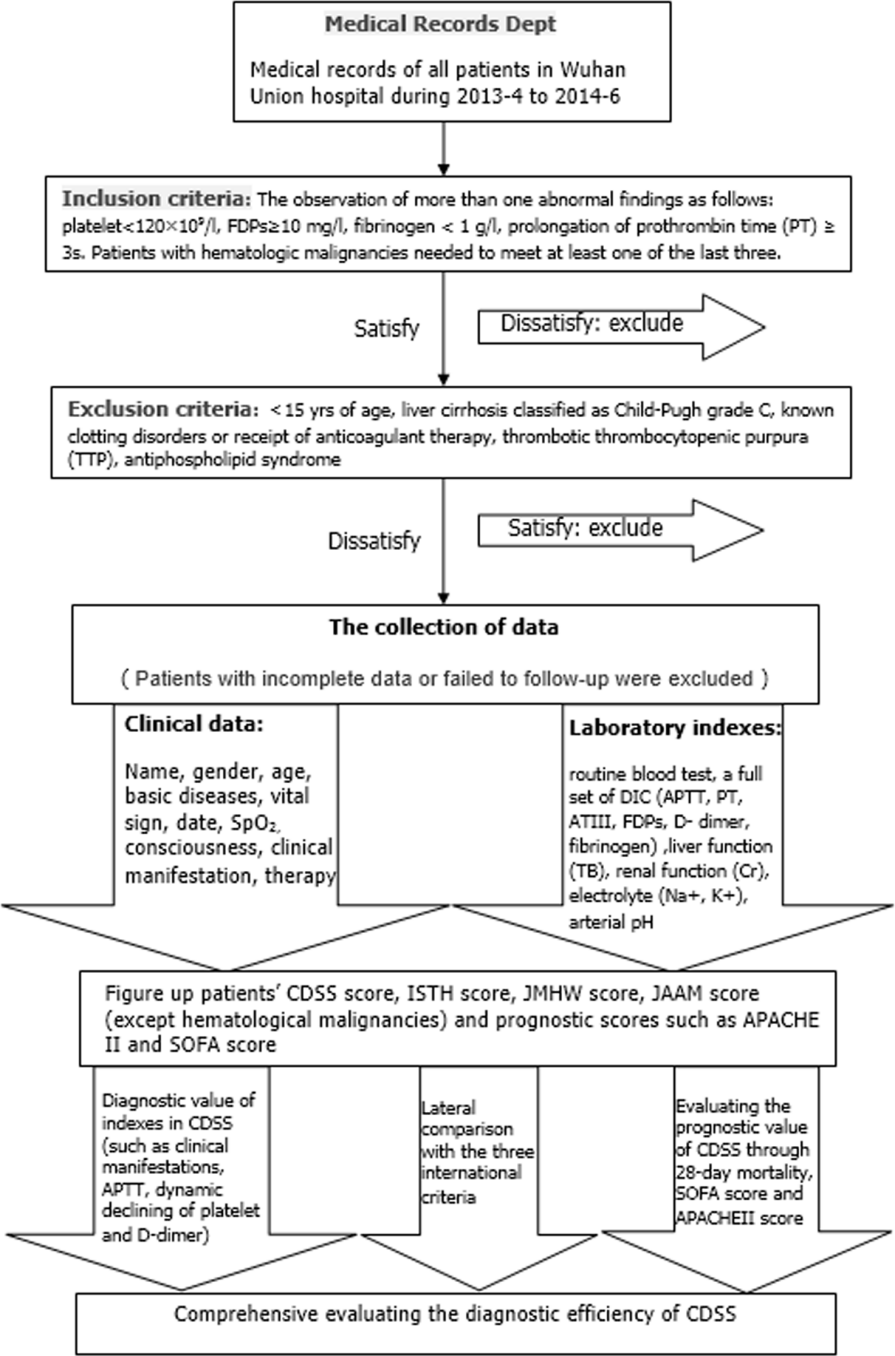
The flow diagram of the whole study process.

Relevant demographic, clinical and laboratory parameters were noted. In order to avoid the bias caused by the repeated collections of a single patient, each case was collected only once at the most severe stage. We also emphasized the objectivity of data and the time consistency between clinical symptoms and laboratory tests. All patients were followed up for 28 days after enrollment in this study, and 28-day mortality was assessed. D-Dimer, FDP, PT, APTT and fibrinogen were tested by STA-R Evolution analyzer (Stago, France). All the laboratory tests were completed in the Clinical Detection Department of Union Hospital, using the same instruments and unified reagents. Management of DIC included transfusion of blood products along with standard treatment of the underlying disorders [3].The flow diagram of this study is shown in Fig 1.

### Statistical Analysis

All measurements were expressed as the mean ± standard deviation (SD). Comparisons between two groups were made with unpaired Student’s t-test or variance analysis for parametric data. Proportions were compared by the chi-square test or Fisher’s exact test when necessary. Receiver operating characteristic (ROC) curves were constructed to assess the diagnostic efficacy and prognostic prediction of each criterion. The areas under the ROC curve (AUC) with standard error (SE) were examined by a significance test for AUC. The relationships between dependent and independent variables were analyzed using a stepwise logistic regression analysis with backward elimination based on the likelihood ratio. Results were reported as odds ratios (ORs) and 95% confidence intervals (CIs). The IBM SPSS18.0 and Medcalc v12.3.0.0 software program were used for the statistical analyses and calculations. Differences with a p value<0.05 were considered to be statistically significant.

## Results and Discussion

### Characteristics of CDSS

#### The values of the clinical manifestations, the dynamic declining platelet count and activated partial thromboplastin time (APTT) in DIC diagnosis.

**Fig 2:**
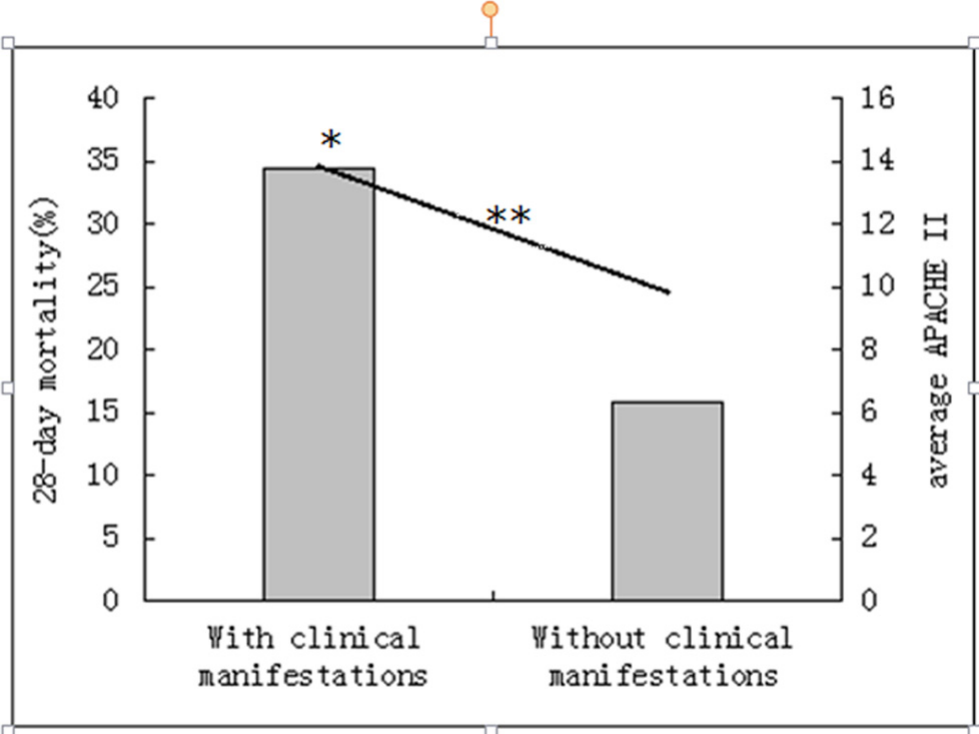
Acute Physiology and Chronic Health Evaluation II (APACHE II) score and 28-day mortality by the clinical manifestation. Bar graph shows 28-day mortality (*p = 0.000). Line graph shows the average APACHE II score (**p = 0.000).

**Fig 3:**
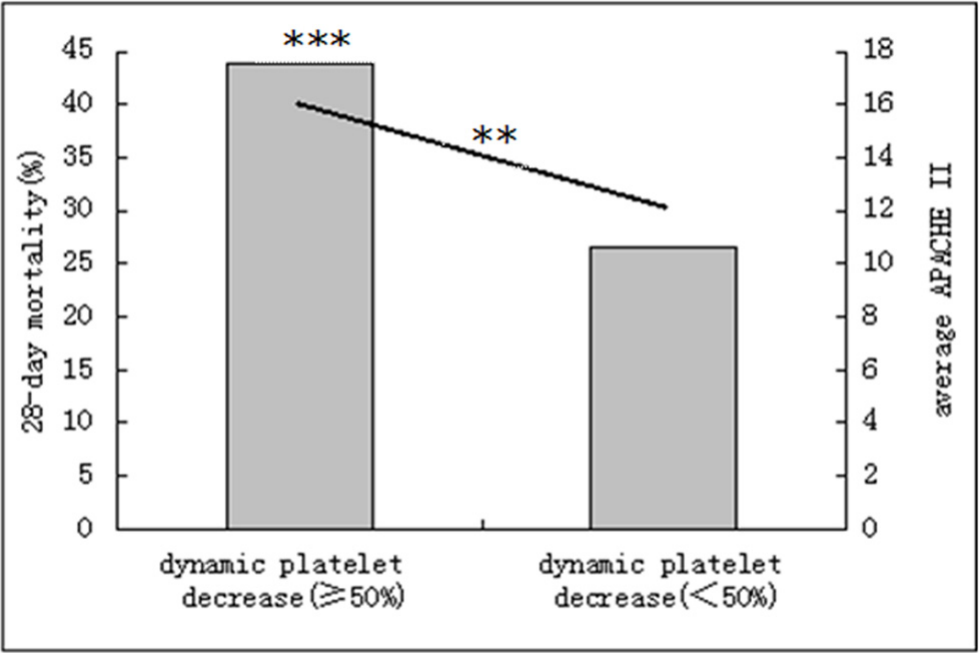
Acute Physiology and Chronic Health Evaluation II (APACHE II) score and 28-day mortality by the dynamic platelet decrease. Bar graph shows 28-day mortality (***p = 0.003). Line graph shows the average APACHE II score (**p = 0.000).

**Fig 4:**
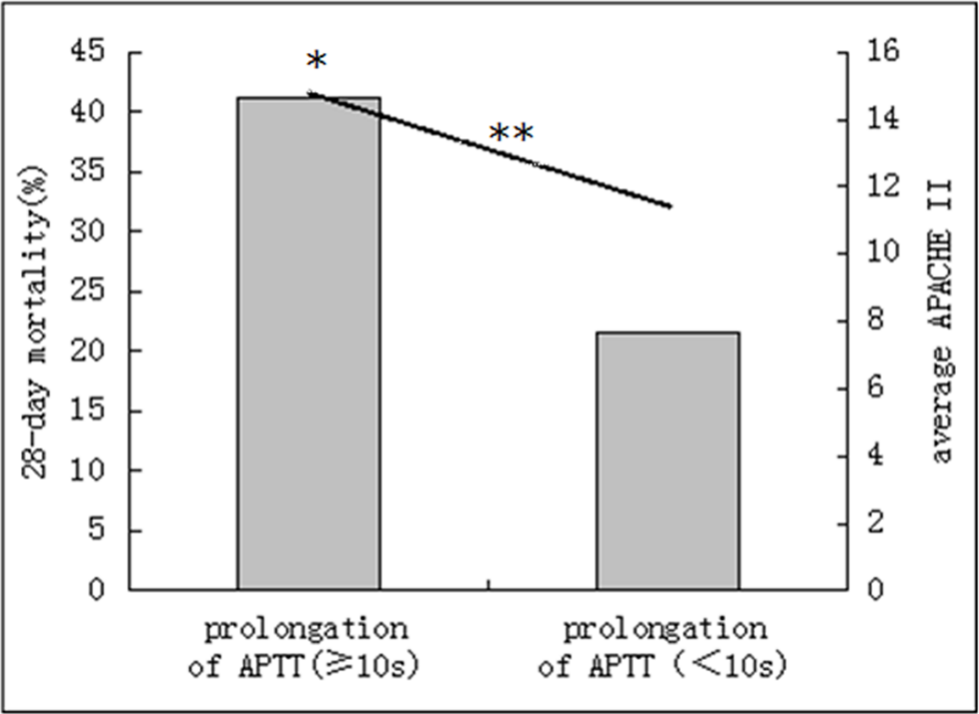
Acute Physiology and Chronic Health Evaluation II (APACHE II) score and 28-day mortality by prolongation of activated partial thromboplastin time (APTT). Bar graph shows 28-day mortality (*p = 0.000). Line graph shows the average APACHE II score (**p = 0.000).

In patients with the clinical manifestations or the declining platelet count (a decrease of ≥50% per 24 hrs) or the prolongation of APTT(≥10s), The 28-day mortality rate, SOFA scores or APACHE II scores were significantly greater than those without (P < 0.01), the scores of CDSS and the DIC diagnostic rate also significantly greater (P < 0.01) (Figs 2, 3 and 4).

The clinical manifestations had a significant impact on the diagnosis of DIC in the multiple logistic regression analysis when using ISTH criteria and JMHW criteria as the standard respectively (OR, 1.936(1.124,3.337), P = 0.017 in ISTH; OR, 17.411(7.283,41.619), P = 0.000 in JMHW).

The dynamic declining platelet count and APTT had a significant impact on death in the multiple logistic regression analysis when using the 28-day mortality as the standard(OR, 2.090(1.155,3.781), P = 0.015 for the declining platelet count; OR, 1.011(1.002,1.021), P = 0.015 for APTT).

#### Using D-dimer as the fibrin-related maker

D-Dimer had a significant impact on the diagnosis of DIC in the multiple logistic regression analysis when using ISTH criteria and JMHW criteria as the standard respectively (OR, 1.221(1.171,1.273), P = 0.000 in ISTH; OR, 1.269(1.211,1.329), P = 0.000 in JMHW), but FDP didn’t.

#### The critical values of platelets and D-dimer

In non-hematologic malignancies, the cut-offs of platelets were approximately 100×10^9^/l when using JAAM as the standard and 80×10^9^/l when using ISTH, JMHW and the 28-days mortality respectively. Because of the highest sensitivity of JAAM, the critical value (100×10^9^/l) was larger for the early diagnosis of DIC. In summary, taking both sensitivity and specificity into account, we took 80×10^9^/l and 100×10^9^/l as the critical values of platelets.

In hematologic malignancies, the cut-off of platelets was approximately 50×10^9^/l when using ISTH and the 28-day mortality as the standard respectively, so we took 50×10^9^/l as the critical value of platelets.

In all patients, the cut-offs of D-dimer were approximately 5mg/l when using ISTH and JMHW as the standard respectively and 9mg/l when using JAAM. Because of the highest sensitivity of JAAM, the critical value (9mg/l) was larger for the early diagnosis of DIC. In summary, taking both sensitivity and specificity into account, we took 5mg/l and 9mg/l as the critical values for D-dimer.

### Evaluating the diagnostic efficacy for DIC in patients with non-hematologic malignancies

#### Baseline Characteristics of the Patients

We retrospectively collected the information of 371 patients who suspected of DIC during 2013–6 to 2014–2. They consisted of 192 males (51.75%, aged 58.30±16.52 years) and 179 females (48.25%, aged 48.60±18.79 years). Underlying diseases included infection, trauma/post-surgery/poisoning, solid tumors, autoimmune diseases, obstetric complications, shock/hypoxic-ischemic, vascular anomalies, severe liver disease (unreached Child-Pugh C grade) and others (Table 2).

**Table 2:** Classification of underlying diseases and diagnostic rate by each criteria for DIC.

Underlying diseases	ISTH	JMHW	JAAM	CDSS	Number (%)
Infection (%)	71(48.97%)	85(58.62%)	111(76.55%)	86(59.31%)	145(23.42%)
Solid tumors (%)	14(43.75%)	18(56.25%)	21(65.63%)	20(62.50%)	32(5.17%)
Autoimmune diseases (%)	5(35.71%)	8(57.14%)	10(71.43%)	9(64.29%)	14(2.26%)
Trauma/post-surgery/poisoning (%)	39(38.24%)	46(45.10%)	69(67.65%)	54(52.94%)	102(16.48%)
Obstetric complications (%)	7(28.00%)	9(36.00%)	15(60.00%)	13(52.00%)	25(4.04%)
Vascular anomalies (%)	4(57.14%)	3(42.86%)	5(71.43%)	4(57.14%)	7(1.13%)
Shock/hypoxic-ischemic (%)	16(72.73%)	16(72.73%)	19(86.36%)	19(86.36%)	22(3.55%)
Severe liver diseases (%)	3(37.50%)	4(50.00%)	5(62.50%)	3(37.50%)	8(1.29%)
Hematologic malignancies (%)	122(49.19%)	130(52.42%)	—-	122(49.19%)	248(40.06%)
Unknown reason (%)	—-	5(31.25%)	—-	4(25.00%)	16(2.58%)

DIC, disseminated intravascular coagulation; ISTH, International Society on Thrombosis and Haemostasis; JMHW, Japanese Ministry of Health and Welfare; JAAM, Japanese Association for Acute Medicine; CDSS, Chinese DIC scoring system.

#### Evaluation of the diagnostic performance

**Fig 5:**
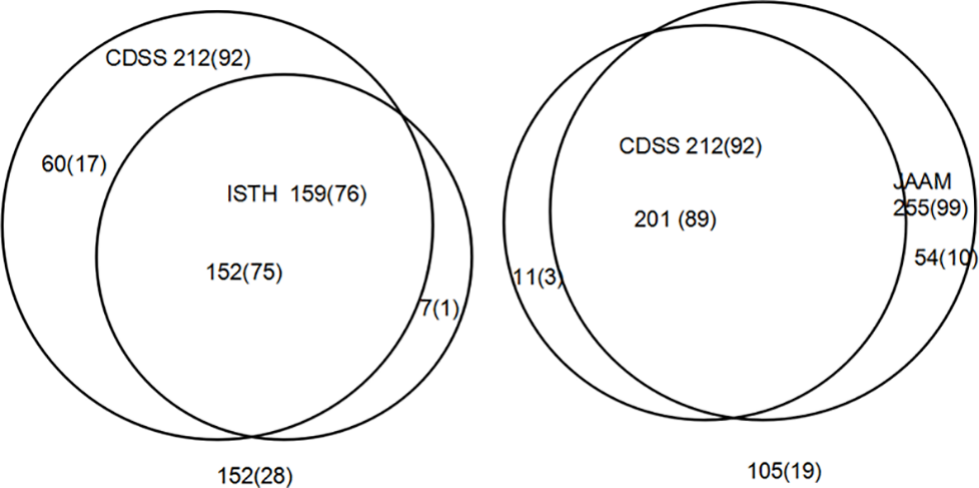
Distribution of patients with non-hematological malignancy according to the three diagnostic criteria. Left, comparison between the International Society on Thrombosis and Haemostasis (ISTH) and Chinese DIC scoring system (CDSS); right, comparison between the Japanese Association for Acute Medicine (JAAM) and Chinese DIC scoring system (CDSS). Numbers in parentheses are of nonsurvivors.

In these 371 patients, the prevalence of DIC by CDSS, ISTH, JAAM, JMHW were 57.14%, 42.86%, 68.73%, 52.29%, respectively. JAAM was the highest (p<0.01) and ISTH was the lowest(p<0.05)(Fig 5). Infection, trauma\ post-surgery\ poisoning and tumor (including hematological malignancies) had the most cases diagnosed as DIC. In patients diagnosed by CDSS, ISTH, JAAM, and JMHW, the 28-day mortality rates were 43.40%, 47.80%, 38.82%, 43.81% respectively(p>0.05). JAAM had the highest sensitivity for death (81.82%), while ISTH had the highest specificity for death (66.80%). The odds ratio (95% confidence interval) for death was 3.44(2.14, 5.53) in CDSS, 3.40(2.18, 5.30) in ISTH, 2.71(1.62, 4.55) in JAAM and 3.01 (1.91, 4.74) in JMHW (Table 3).

**Table 3:** Characteristics of the diagnosis and mortality by each criterion in non- hematological malignancy.

	ISTH	JMHW	JAAM	CDSS
Diagnostic ratio of DIC	42.86%^a^	52.29%^b^	68.73%^a^	57.14%
28-day mortality	47.80%^b^ ^,^ ^c^	43.81%^b^	38.82%^b^	43.40%
Sensitivity of death	62.81%^a^	70.25%^b^	81.82%^b^	76.03%
Specificity of death	66.80%^a^	56.40%^b^	37.60%^a^	52.00%
AUC*	0.671	0.675	0.675	0.682
OR (95%CI)^#^	3.40(2.18,5.30)	3.01(1.91,4.74)	2.71(1.62,4.55)	3.44(2.14,5.53)

ISTH, International Society on Thrombosis and Haemostasis; JMHW, Japanese Ministry of Health and Welfare; JAAM, Japanese Association for Acute Medicine; CDSS, Chinese DIC scoring system; DIC, disseminated intravascular coagulation; AUC, area under receiver operating characteristic curve; OR, Odds ratio; CI, confidence interval.

a:p<0.05 for CDSS compared with other criteria;

b:p>0.05 for CDSS compared with other criteria;

c:P>0.05 between JAAM and ISTH;

*P>0.05 between any two;

#p<0.05 for each OR.

#### ROC curve analysis

Using ISTH as the standard, the sensitivity and the specificity of CDSS and JAAM were 93.41% vs. 98.80% (p<0.05) and 72.55% vs. 50.49% (p<0.05) respectively. The AUC of CDSS and JAAM were 0.925 and 0.930 respectively(p>0.05).

Using JAAM as the standard, the sensitivity and the specificity of CDSS and ISTH were 77.07% vs. 62.03% (p<0.05) and 93.33% vs. 98.10% (p = 0.088). The AUC of CDSS and ISTH were 0.930 and 0.912 respectively (p>0.05).

Using the 28-day mortality as the standard, the AUC of CDSS, ISTH, JAAM, JMHW were 0.682, 0.671, 0.675, 0.675 (p>0.05 between any two).

### Evaluating the diagnostic efficacy for DIC in patients with hematologic malignancies

#### Baseline Characteristics of the Patients

We retrospectively collected the information of 248 patients who suspected of DIC during 2013–4 to 2014–6. They consisted of 150 males (60.48%, aged 45.63±18.33 years) and 98 females (39.52%, aged 46.18±15.36 years). Underlying diseases included AML-M3 (37 cases, 14.92%), AML except M3 (92 cases, 37.10%), ALL (44 cases, 17.74%), CL (21 cases, 8.47%), lymphoma (23 cases, 9.27%), MM (14 cases, 5.65%), MDS (12 cases, 4.84%) and others (5 cases, 2.02%).

#### Evaluation of the diagnostic performance

**Fig 6:**
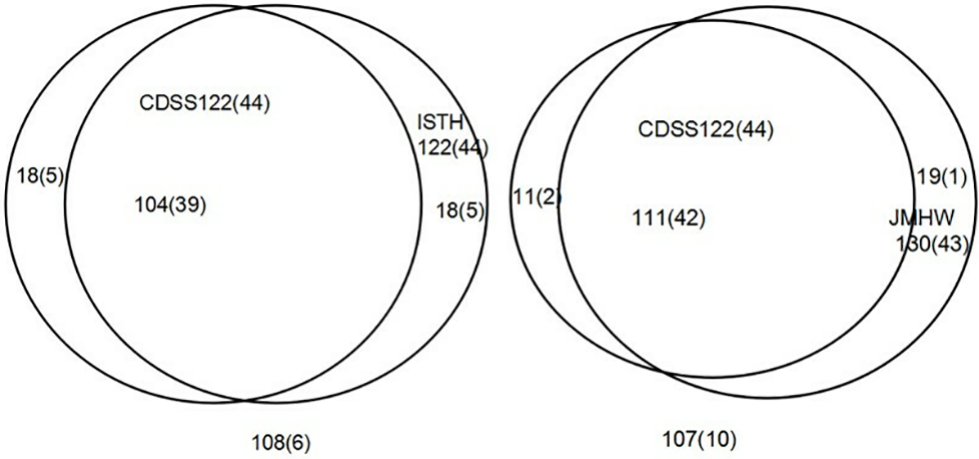
Distribution of patients with hematological malignancy according to the three diagnostic criteria. Left, comparison between the International Society on Thrombosis and Haemostasis (ISTH) and Chinese DIC scoring system (CDSS); right, comparison between the Japanese Ministry of Health and Welfare (JMHW) and Chinese DIC scoring system (CDSS). Numbers in parentheses are of nonsurvivors.

In these 248 patients, the prevalence of DIC by CDSS, ISTH, JMHW were 49.19%, 49.19%, 52.42% respectively(p>0.05). JMHW criteria had the highest diagnostic rate (Fig 6). AML, ALL, lymphoma had the most cases diagnosed as DIC, especially APL with the highest morbidity (75.68%). In patients diagnosed by CDSS, ISTH and JMHW, the 28-day mortality rates were 36.07%, 36.07%, 33.08% respectively(p>0.05). CDSS and ISTH had the higher sensitivity(80.00%, p>0.05) and specificity (59.59%, p>0.05)for death. The odds ratio (95% confidence interval) for death was 5.90(3.01, 11.56) in CDSS, 5.90(3.01, 11.56) in ISTH and 4.37(2.24, 8.52) in JMHW (Table 4).

**Table 4:** Characteristics of the diagnosis and mortality by each criterion in hematological malignancy.

	ISTH	JMHW	CDSS
Diagnostic ratio of DIC	49.19%	52.42%^a^	49.19%
28-day mortality	36.07%	33.08%^a^	36.07%
Sensitivity of death	80.00%	78.18%^a^	80.00%
Specificity of death	59.59%	54.92%^a^	59.59%
AUC*	0.759	0.730	0.769
OR (95%CI)#	5.90(3.01,11.56)	4.37(2.24,8.52)	5.90(3.01,11.56)

ISTH, International Society on Thrombosis and Haemostasis; JMHW, Japanese Ministry of Health and Welfare; CDSS, Chinese DIC scoring system; DIC, disseminated intravascular coagulation; AUC, area under receiver operating characteristic curve; OR, Odds ratio; CI, confidence interval.

a:p>0.05 for CDSS compared with other criteria;

*: P>0.05 between CDSS and ISTH, P<0.05 between CDSS and JMHW;

#p<0.05 for each OR.

#### ROC curve analysis

Using ISTH as the standard, the sensitivity and specificity of CDSS and JMHW were 85.25% vs. 83.61% (p = 0.724) and 85.71% vs. 77.78% (p = 0.103). The AUC of CDSS and JMHW were 0.933 and 0.889 respectively(p<0.01).

Using JMHW as the standard, the sensitivity and specificity of CDSS and ISTH were 85.38% vs. 78.46% (p = 0.147) and 90.68% vs. 83.05% (p = 0.083). The AUC of CDSS and ISTH were 0.944 and 0.845 respectively(p<0.01).

Using the 28-day mortality as the standard, the AUC of CDSS, ISTH, JMHW were 0.769, 0.759, 0.730(p<0.05 between CDSS and JMHW, p>0.05 between CDSS and ISTH).

### Evaluating the prognostic value of CDSS in DIC patients

**Fig 7:**
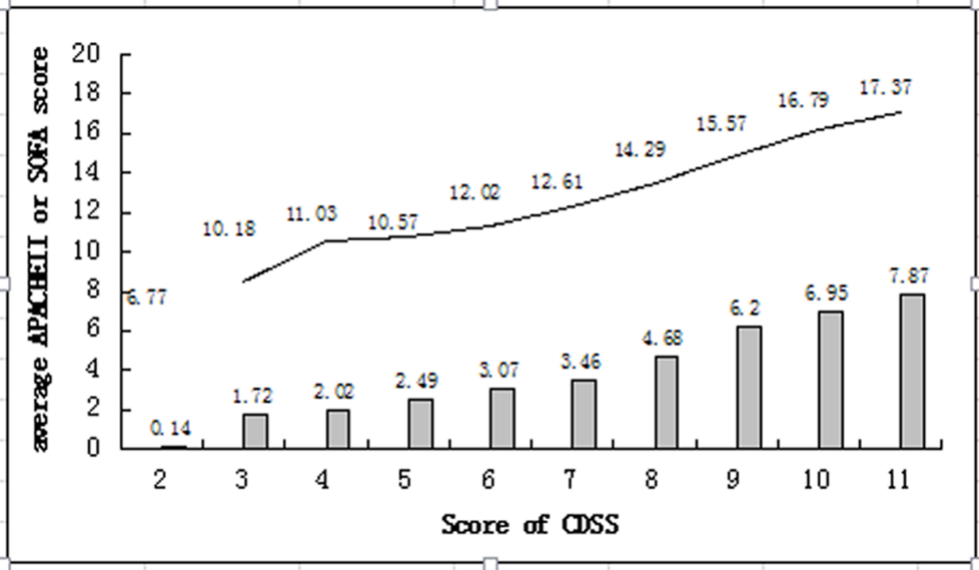
Relativity between CDSS scores and average APACHE II or SOFA scores. CDSS, Chinese DIC scoring system; APACHE, Acute Physiology and Chronic Health Evaluation; SOFA, Sequential Organ Failure Assessment. Bar graph shows average SOFA score. Line graph shows average APACHE II score.

The 28-day mortality rate, SOFA scores, APACHEII scores of DIC patients diagnosed by CDSS were significantly greater than non-DIC (p <0.05) (Table 5). With the increase of CDSS scores, APACHEII scores and SOFA scores increased simultaneously (Fig 7).

**Table 5:** Characteristics of DIC and non-DIC patients diagnosed by CDSS.

	DIC	Non-DIC	P value
Age (years)	51.19±17.61	49.70±18.95	0.311
Gender (male/female)	186/148	156/129	0.812
Disease distribution*	86/20/9/54/13/4/ 19/3/4/122	59/12/5/48/12/3/3/5/12/126	0.012
Platelet count(10^9^/L)	57.33±70.03	122.93±112.65	<0.01
D-Dimer (mg/L)	13.81±6.07	6.03±5.30	<0.01
FDP(mg/L)	81.95±65.92	30.55±38.54	<0.01
ATA (%)	63.50±27.15	70.88±23.89	<0.01
PT(s)	25.15±18.93	17.73±6.30	<0.01
APTT(s)	62.59±55.10	49.46±23.22	<0.01
Fibrinogen (g/L)	2.48±1.85	3.56±2.00	<0.01
APACHE II score	14.12±7.82	10.80±5.53	<0.01
SOFA score	6.17±5.01	2.94±3.33	<0.01
28-day mortality	40.72%(136/334)	14.04%(40/285)	<0.01
Decline of PLT (%)	51(15.27%)	15(5.26%)	<0.01
With clinical manifestations (%)	271(81.14%)	152(53.33%)	<0.01

*: sort order (infection, solid tumors, autoimmune diseases, trauma/post-surgery/poisoning, obstetric complications, vascular anomalies, shock/hypoxic-ischemic, severe liver disease, unknown reason, hematological malignancy); CDSS, Chinese DIC scoring system; DIC, disseminated intravascular coagulation; APACHE, Acute Physiology and Chronic Health Evaluation; SOFA, Sequential Organ Failure Assessment; PLT, Platelet counts; APTT, activated partial thromboplastin time; PT, Prothrombin time; FDP, fibrin/fibrinogen degradation products; ATA, Antithrombotic Agents.

## Discussion

The diagnosis of DIC is still a challenge. Good diagnostic criteria should have the appropriate sensitivity, specificity and be easy to use. In 1986, China had proposed its first DIC diagnostic criteria and modified it several times with time going. Along with the appearance of ISTH criteria in 2001 and JAAM criteria in 2006, we have gradually realized that a scoring system may be more favorable for DIC diagnosis. So we proposed CDSS based on the latest 2012 Chinese experts’ consensus on DIC diagnosis.

CDSS has the following characteristics: 1> It takes the underlying diseases as one of its integral items instead of the necessary precondition in DIC diagnosis. In clinical practice, we found that a few patients didn’t manifested obvious disorders related to DIC when developing DIC. This change may improve the DIC diagnostic sensitivity. 2> it emphasizes the role of the clinical manifestations in DIC diagnosis: The clinical manifestations are important clues for doctors, and also one of the characteristics of DIC [20]. In this study, we can see that the clinical manifestations had a significant impact on the DIC diagnosis and prognosis. 3> it emphasizes the value of the dynamic declining platelet count in DIC diagnosis: In the early state of diseases or after the infusion therapy, the absolute platelet count can maintain in the normal range, but the dynamic decrease prompts the progressive consumption of platelets. In the study of Gando [14], it showed that due to the dynamic decrease of platelets, the DIC patients increased nearly 21%. Therefore, the dynamic decrease of platelets can make up for the limitation of absolute platelet count and help to the early diagnosis of DIC. 4> it uses D-dimer as the fibrin-related maker: Theoretically speaking, the elevation of D-dimer often prompts the secondary fibrinolysis [21], which is consistent with the characteristic of DIC [1]. This study showed that D-dimer had a significant impact on DIC diagnosis, but FDPs not. A Meta analysis [22] showed that the area under the summary ROC of D-dimer for DIC diagnosis was greater than FDPs (0.9144vs0.7917). So using D-dimer as the fibrin-related maker may have more benefit than FDPs. 5> it points out the value of APTT in DIC diagnosis: The study showed that APTT had an impact on the DIC diagnosis and prognosis. So we brought APTT in the global coagulation items together with PT. This may improve the diagnostic sensitivity of DIC. 6> it takes account of the specificity of DIC with hematologic malignancies [23]: Due to the influences of the primary disorders and treatments, the absolute platelet count had limited value for this kind of DIC diagnosis. We adjusted the critical value as 50×10^9^/l in this condition. Bleeding is a common manifestation of hematologic malignancies [24–27] (the bleeding ratio was 25% in this study), therefore, we removed the hemorrhage from the clinical manifestations item.

In non-hematologic malignancies study, JAAM had the highest prevalence (68.73%,p<0.01), which consistent with other literatures [9–11]. ISTH had the highest specificity for death (66.80%, p = 0.057 compared with JMHW, p<0.05 with others). JAAM seemed too sensitive and ISTH was relatively strict. This study showed that CDSS was more specific than JAAM(p<0.05) and more sensitive than ISTH(p<0.05). We can see that CDSS lied in the intermediate state, taking both the sensitivity and the specificity into account. This may be conducive to the early diagnosis of DIC while reducing the false positive rate and the waste of medical resources. In addition, areas under the ROC of CDSS were both larger than 0.9 when using JAAM and ISTH as the standard respectively. In hematological malignancies study, the areas under the ROC of CDSS were significantly larger than ISTH and JMHW(p<0.01). These prompted its good diagnostic efficacy of DIC.

Finally, we found that area under the ROC of CDSS was similar to other scoring systems when we used the 28-day mortality as the standard. So we can see that CDSS had a good prognostic value for DIC patients.

Since this study was a single-center, retrospective study, we put efforts into the whole study process including the objective selection of subjects, the data collection and the comprehensive analysis to reduce the bias as far as possible. However, there were still some limitations of this study. First of all, the representation of these objects was limited. In addition, we can’t follow up the conditions of patients dynamically, and lacked of the unified standard in the observation of clinical symptoms. The multicenter, prospective study is needed to further confirm the diagnostic efficacy of CDSS.

## Conclusions

This study showed that, in non-hematologic malignancies, CDSS was more sensitive than ISTH, and more specific than JAAM. In hematologic malignancies, CDSS had higher diagnostic efficacy than other systems. In addition, CDSS had a good prognostic value for DIC patients. In summary, CDSS is suitable for DIC diagnosis, and expected to be a new generation of DIC diagnostic criteria applicable to clinical practice.

This study was a retrospective statistical analysis; conclusions need to be further confirmed by multi-center, prospective study.

## Supporting Information

S1 Dataset The clinical and laboratory’s data of all the subjects. (XLSX)
